# An *In-vivo* Study into the Effects of Schisandrin B in the Liver, Spleen, Kidney, and Brain of Acute Thioacetamide-intoxicated Mice 

**DOI:** 10.22037/ijpr.2021.115154.15225

**Published:** 2021

**Authors:** Ho Yin Pekkle Lam, Meng-Yun Hung, Ting-Ruei Liang, Shih-Yi Peng

**Affiliations:** a *Institute of Medical Sciences, Tzu Chi University, Hualien, Taiwan. *; b *Department of Biochemistry, School of Medicine, Tzu Chi University, Hualien, Taiwan.*; c *Ph.D. Program in Pharmacology and Toxicology, School of Medicine, Tzu Chi University, Hualien, Taiwan.*

**Keywords:** Thioacetamide, Schisandrin B, Hepatotoxicity, Inflammasome, Apoptosis

## Abstract

Currently, there are no effective treatments for liver diseases. Treatment usually involves controlling complications and supportive care. As liver injuries also affect other organs such as the spleen, kidney, and brain due to their anatomical and physiological relationships, finding an effective treatment is urgently needed. This research aimed to evaluate the therapeutic effect of Schisandrin B (Sch B) in the liver and other organs in thioacetamide (TAA)-intoxicated mice. In this study, mice were exposed to a single intraperitoneal injection of 200 mg/kg TAA to induce hepatitis. Following Sch B (20 mg/kg/day, 28 consecutive days) treatment, biochemistry analysis and histopathological examination of different organs were performed, in addition to western blotting and flow cytometry to evaluate the involvement of inflammasomes and apoptotic proteins. Our results showed that administration of Sch B protected against TAA-induced damages, and it disparately affected inflammasome activation and apoptosis in different organs. Furthermore, Sch B treatment improved organ function, as indicated by the improvement of serum biochemical parameters. Collectively, our findings reveal a beneficial effect of Sch B on different organ damages in mice intoxicated with TAA.

## Introduction

Currently, there are no effective treatments for liver diseases. Treatment usually involves controlling complications and supportive care (1). Additionally, liver injuries affect other organs such as the spleen, kidney, and brain due to their anatomical and physiological relationships (2-4). Moreover, the liver carries almost 25% of blood in each cardiac cycle, making the “liver-organs” axis exerts different influences on the organ’s pathogenesis. For example, liver injuries are usually accompanied by splenomegaly because the portal vein links between the two organs (5). Hepatic encephalopathy occurs when the damaged liver can no longer remove toxic substances (6); whereas hepatic nephropathy occurs due to hepatorenal syndrome (7). Therefore, finding an effective treatment for liver disease is urgently needed. 

Two very important mechanisms involved in organ injuries are inflammation and apoptosis. Inflammation initially occurs to protect our tissue from infections and injuries (8, 9). However, excessive inflammation causes unwanted tissue injuries. Inflammation is usually the result of inflammasome activation (9). The inflammasome is an oligomeric complex that detects pathogens-derived or self-derived activation signals and releases inflammatory cytokines (10). Upon recognizing an activation signal, inflammasome sensors including NOD-like receptor-pyrin-containing (NLRP) 1, NLRP3, NLRC4, and NLRP6 will recruit caspase-1, with or without the adaptor protein, apoptosis-associated speck-like protein containing a CARD (ASC) (11). Activated caspase-1 then cleavages pro-inflammatory cytokines interleukin (IL)-1β and IL-18 into their active forms. In addition, caspase-1 triggers gasdermin D (GSDMD) cleavage, which causes pyroptosis (10, 11).

Apoptosis is a form of programmed cell death that leads to cell morphological changes and death (12). Apoptosis mainly follows one of two pathways, extrinsic and intrinsic. The extrinsic pathway involves the activation of cell-surface death receptors triggered by extracellular cell death ligands such as Fas ligand (FasL) and tumor necrosis factor (TNF); whereas the intrinsic pathway originates within the mitochondrion upon cell damages (12, 13). Apoptosis is often caspase-dependent. In the extrinsic pathway, activation of the cell-surface death receptor leads to the recruitment of caspase-8, which in turn activates caspase-3, the chief effector caspases of apoptosis (14, 15). In the intrinsic pathway, cell stress signals may induce mitochondria to release cytochrome c, thereby causing caspase-9-containing apoptosome activation. The apoptosome then recruits and activates caspase-3 (15). Furthermore, activation of caspase-8 in the extrinsic pathway can result in the cleavage of the pro-apoptotic protein Bid, which sequentially translocates to the mitochondria to release cytochrome c (15).

Schisandrin B (Sch B), the most abundant active ingredient in *Schisandra chinensis*, has shown a protective effect against liver injuries, including fatty liver disease (16, 17), drug-induced hepatotoxicity (18-20), hepatic fibrosis (21), and even hepatoma (22). Additionally, Sch B has shown a beneficial role in chemical-induced tissue injuries and organ damages. Examples such as mercury-induced renal damage (23), paraquat-induced neuronal damage (24), and doxorubicin-induced cardiotoxicity (25) provided further confirmation on the beneficial role of Sch B in tissue injuries. 

Thioacetamide (TAA) administration has shown to induce injuries in the liver (26, 27), as well as the spleen (28), kidney (29), and brain (30) due to the “liver-organs” axis. In the current study, we strove to investigate the beneficial effect Sch B has on different organs including the liver, spleen, kidney, and brain in mice exposed to a single, high dose TAA. 

## Experimental

*Schisandrin B*

Schisandrin B (Sch B; C_23_H_28_O_6_, molecular weight 400.47 kDa, isolated from *Schisandra chinensis* with purity exceeding 98% as assessed by HPLC) was purchased from Chengdu Alfa Biotechnology (Chengdu, China). Sch B was dissolved in olive oil to yield a concentration of 20 mg/kg. 

*Animal treatment*

**Figure 1:**
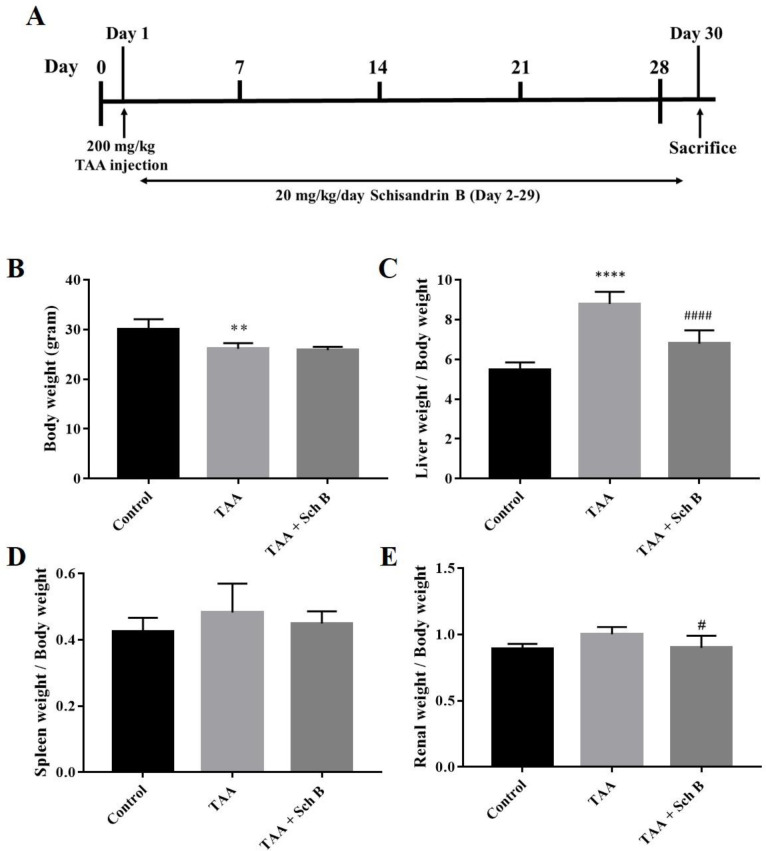
Treatment scheme of the study, body weight, and organs weight. (A) Mice were injected 200 mg/kg TAA intraperitoneally. One day later, mice were treated either with 20 mg/kg/day Sch B or olive oil (vehicle) for 28 consecutive days. (B) Body weight. (C-E) Liver, spleen, and kidney weight index. Results were expressed as mean ± SD (n = 10). ^*^P-value < 0.05, ^** ^*P*-value < 0.01, ^**** ^*P*-value < 0.0001 compared with control; ^##^*P*-value < 0.01, ^####^*P*-value < 0.0001 compared with TAA

Male BALB/c mice (National Laboratory Animal Center, Taipei, Taiwan), aged 6-8 weeks, were divided into three groups—control group, the TAA group, and TAA+Sch B group. Mice from the TAA and TAA+Sch B group were treated with a single intraperitoneal injection of 200 mg/kg TAA (Alfa Aesar, Ward Hill, MA, USA) to induce acute hepatitis (26). One day later the mice in the TAA+Sch B group were treated with 20 mg/kg Sch B by oral gavage for 28 consecutive days (31, 32). Mice from the control and TAA groups were treated with olive oil (Sch B vehicle) using the same procedures. All mice were sacrificed on day 30 (Figure 1A). Upon dissection, liver, spleen, kidney, and brain tissues were collected. All experimental protocols involving animals were reviewed and approved by the IACUC of Tzu Chi University (No. 109066).

*Hematoxylin & eosin (H&E) staining*

Samples were fixed in 10% neutral buffered formalin and dehydrated in a series of graded dilutions of alcohols. After immersion in xylene and molten paraffin, the tissues were cut into thin sections. Slides were then stained as follows: hematoxylin, water, acid alcohol, water, eosin, 95% ethanol, and 100% ethanol (31). 

*Histopathological examination and scoring*

Five sections from each group were examined. Ten random fields were examined and scored in each section. Histopathological changes in the liver were evaluated as follows: 0, no evidence of injuries; 1, mild injuries with cytoplasmic vacuolation and pyknosis; 2, moderate to severe injuries with extensive pyknosis and loss of intercellular borders; and 3, severe necrosis with the disintegration of hepatic cords, congestion, and infiltration (27). Spleen sections were scored based on the following three criteria: enlargement of lymphocyte areas (0, absent; 1, mild; 2, moderate; and 3, pronounced); the presence of germinal centers (0, present; and 1, absent); and presence of apoptosis, necrosis, pigments, or macrophages (0, absent and 1, present) (33). Scores from the three criteria were summated to give a total score of 5 (0, no injuries; 5, severe injuries). For kidney sections, the score was given based on the presence of tubular necrosis, loss of brush border, cast formation, and tubular dilatation according to 0, none; 1, ≤10%; 2, 11–25%; 3, 26–45%; 4, 46–75%; and 5, >76% (34). Brain sections were scored as 0, no evidence of injury; 1, rare but dispersed pyknosis; 2, single or small confluent areas of necrosis or apoptosis; 3, large or multiple areas of necrosis or apoptosis (35).

*RNA isolation, cDNA synthesis, and real-time quantitative PCR*

Splenic RNA extraction, cDNA synthesis, and real-time quantitative PCR (qPCR) were performed as described previously (31). The sequences of primers used are shown in Table 1. The ΔΔCt method was used to analyze the relative gene expression normalized to *β-actin*. 

*Protein extraction and western blotting*

Total protein was extracted from tissues using 1 mL RIPA buffer (Thermo Scientific, Rockford, IL, USA). Samples were resolved by SDS-PAGE and transferred to a PVDF membrane (EMD Millipore, Burlington, MA, USA). After blocking, membranes were incubated with antibodies against α-tubulin (GeneTex, Irvine, CA, USA), NLRP3 (Proteintech, Chicago, IL, USA), caspase-1 (Proteintech), IL-1β (Cell Signaling Technology, Danvers, MA, USA), IL-18 (Proteintech), GSDMD (Santa Cruz Biotechnology, Dallas, TX, USA), caspase-3 (ABclonal, Woburn, MA, USA), caspase-8 (ABclonal), caspase-9 (ABclonal), and BCL-2 (GeneTex). The membranes were incubated with the HRP-conjugated anti-mouse or anti-rabbit IgG secondary antibodies (EMD Millipore) for 1 h. Protein bands were visualized with ECL reagents (EMD Millipore) and quantified by densitometry using Image J software (v1.46, NIH, Bethesda, MD, USA). All protein bands were normalized by α-tubulin.

*Collection of serum and CSF, and measurement of biochemical parameters*

Cardiac puncture was performed to collect whole blood from the mice. The blood was centrifuged at 600 ×g for 15 min to obtain the serum. CSF was obtained by rinsing the cranial cavity and cerebral ventricles with sterile PBS. The washing solution was collected and centrifuged at 600 ×g for 10 min, and then at 10,000 ×g for 30 min. The supernatant contains CSF along with PBS, which is also called ‘CSF-like fluid’ (31). Serum or CSF was analyzed for different biochemistry parameters using an automated Hitachi 7080 chemistry analyzer (Hitachi Ltd., Tokyo, Japan). 

*Preparation of cell suspension from tissues and determination of apoptosis using flow cytometry*

Liver, kidney, and spleen samples were teased with forceps and needles into phosphate-buffered saline (PBS) supplemented with 5% fetal bovine serum (FBS; Gibco; Thermo Fisher Scientific, Inc.). The cells were filtered through a 40 μm cell strainer (36). Brain samples were dissociated using 0.25% trypsin-EDTA (GeneDireX, Taiwan) and incubated at 37 °C for 10 min. Gentle pipetting was performed to break up tissue clumps. The cells were then filtered through a 40 μm cell strainer (31). All filtered cells were centrifuged for 5 min at 250 ×g and resuspended in ice-cold binding buffer. Cells were then stained with Alexa Fluor Annexin V/Dead Cell apoptosis kit (Molecular Probes Inc., Eugene, OR, USA) and subjected to flow cytometric analysis.

*Statistical analysis*

All results obtained in this study were analyzed by GraphPad Prism 6.01 software (GraphPad Software, San Diego, CA, USA), and presented as mean ± standard deviation. Statistical comparison was done by one-way ANOVA with a post hoc test. A *P*-value less than 0.05 indicates statistical significance.

## Results

*Histopathology and functional status of the liver*

**Figure 2:**
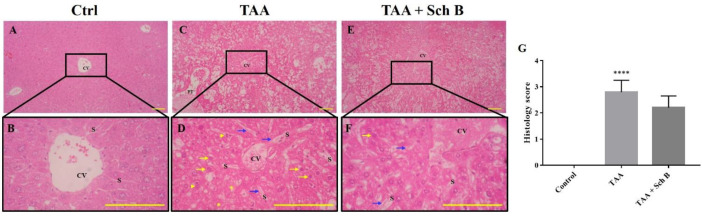
Histopathology of the liver. (A and B) Representative photomicrographs of H&E-stained liver sections from control mice showed normal hepatic architecture. (C and D) Liver sections from mice treated with TAA showed distorted hepatic architecture with congestion of the central vein. In addition, sinusoidal dilatation and congestion (blue arrows), ballooning degeneration (yellow asterisks), hepatocyte apoptosis or pyknosis (yellow arrows), and karyorrhexis (yellow arrowheads) were observed. (E and F) Liver sections from TAA-injected mice treated with Sch B showed fewer pathological hepatocytes and more healthy hepatocytes with normal histology. Images are shown at 40× and 100× magnifications; scale bars, 200 μm. CV, central veins; S, sinusoids; PT, portal triads. (G) Histology score of liver sections, shown as mean ± SD (n = 5). ^****^P-value < 0.0001 compared with control

A single injection of 200 mg/kg TAA has caused significant pathological changes in the mouse liver. The liver weight significantly increased after TAA injection (Figure 1C). Histological study revealed distorted lobular architecture and congestion. In addition, sinusoidal dilation, ballooning degeneration, and hepatocyte apoptosis, pyknosis, or karyorrhexis were noted (Figures 2C-2D and 2G). The functional status of the liver was also altered by TAA, as shown by the increased level of serum alanine transaminase (ALT), aspartate transaminase (AST), and lactate dehydrogenase (LDH); and decreased serum albumin (Table 2). After treatment with Sch B, the liver showed much improvement in the histopathology, indicated with less congestion and more healthy hepatocytes (Figure 2E-2G) and an improved functional status (Table 2). 

*Histopathology of the spleen*

**Figure 3:**
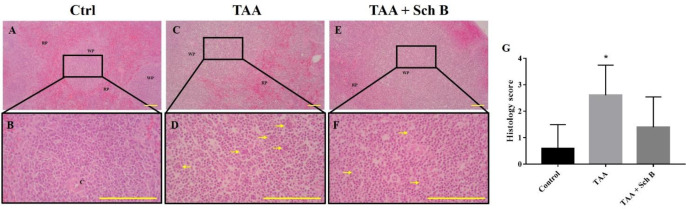
Histopathology of the spleen**. **(A and B) Representative photomicrographs of H&E-stained spleen sections from control mice. (C and D) Spleen sections from TAA-injected mice. White pulps and red pulps were distinguishable, but the junction was not clear. In addition, white pulps were not structurally organized. A moderate decrease of white pulp elements was noted, and tingible body macrophages containing apoptotic bodies (yellow arrowhead) were seen. (E and F) Spleen sections from TAA-injected mice treated with Sch B. Improved structure with fewer apoptotic bodies were seen. Images are shown at 40× and 100× magnifications; scale bars, 200 μm. RP, red pulps; WP, white pulps; C, central artery. (G) Histology score of spleen sections, shown as mean ± SD (n = 5). ^*^P-value < 0.05 compared with control

TAA-intoxicated mice showed a slight increase in spleen weight (Figure 1D). Spleen sections from the TAA group showed white pulps with indistinguishable germinal center and marginal zone. In addition, tingible body macrophages containing apoptotic bodies were seen throughout the white pulps (Figures 3B,3E and 3G). Spleen sections from the TAA+Sch B group showed an improved histological structure with fewer apoptotic cells (Figures 3C, 3F and 3G).

*Histopathology and functional status of the kidney*

**Figure 4:**
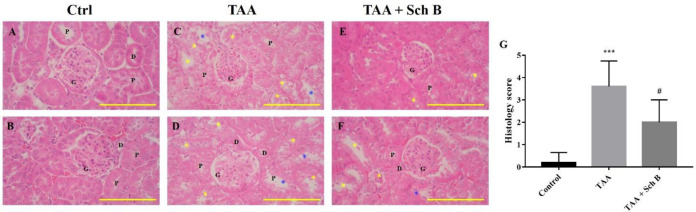
Histopathology of the kidney. (A and B) Two representative photomicrographs of H&E-stained kidney sections from control mice. (C and D) Kidney sections from TAA-injected mice. Degeneration of renal tubular (yellow asterisks) and desquamation (blue asterisks) were seen. (E and F) Kidney sections from TAA-injected mice following Sch B treatment. Fewer tubular degeneration and desquamation were seen. Images are shown at 100× magnifications; scale bars, 200 μm. G, glomeruli; P, proximal convoluted tubules; D, distal convoluted tubules. (G) Histology score of kidney sections, presented as mean ± SD (n = 5). ^***^P-value < 0.001 compared with control; ^#^*P*-value < 0.05 compared with TAA

Renal tubule degeneration, characterized by different morphologies including cell swelling, cytoplasmic vacuolation, and pale-staining cytoplasm, was observed in kidney sections of TAA-injected mice. In addition, desquamation and loss of brush border were seen in tubular epithelial cells (Figures 4C, 4D and 4G). Although these pathological findings were also seen in mice treated with Sch B, the symptoms were much improved (Figures 4E-4G). Parallel with the pathology observed, TAA-injected mice showed an increased level of serum creatinine and blood urea nitrogen (BUN), suggesting an impaired kidney function. All of these serum biochemistry parameters decreased in mice treated with Sch B (Table 2). 

*Histopathology and functional status of the brain*

**Figure 5:**
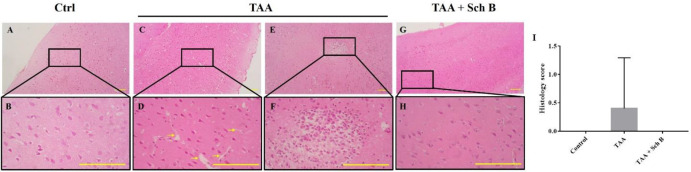
Histopathology of the brain. (A and B) Representative photomicrographs of H&E-stained brain sections from control mice. (C and F) Two representative photomicrographs of H&E-stained brain sections from TAA-intoxicated mice. (C and D) A mild increase of perivascular spaces was seen (yellow arrows). (E and F) One marked area of perivascular infiltration of macrophages and neutrophils was seen in one of the five sections. (G and H) Brain sections from Sch B treated mice. No specific pathological changes. Images are shown at 40× and 100× magnifications; scale bars, 200 μm. (I) Histology score of brain sections, presented as mean ± SD (n = 5).

A mild increase of perivascular spaces was seen in TAA-intoxicated mice (Figure 5B). Only one brain section from the TAA group showed a marked area of perivascular infiltration and hemorrhage (Figures 5E and 5F). Except for this, no other specific pathological changes were seen. We as well investigated the permeability of the blood-brain barrier (BBB) as it separates the brain from peripheral blood and maintains CNS homeostasis (37); therefore, we calculated the CSF/serum total protein and albumin ratio. Our data showed that TAA injection causes a compromised BBB, as indicated by increased albumin and total protein ratio. Following Sch B treatment, both the ratio of total protein and albumin decreased (Table 2). 

*Analysis of inflammasome and apoptosis in the liver*

**Figure 6:**
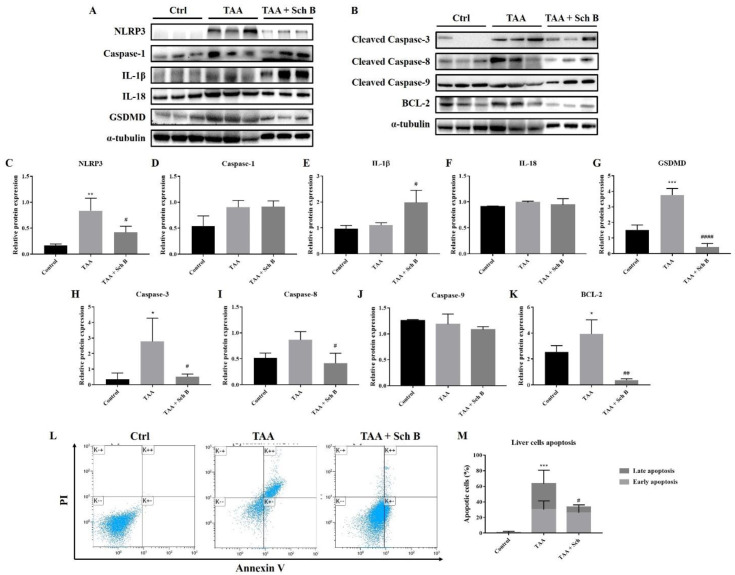
Analysis of inflammasome and apoptosis in the liver. (A) Western blot images showing inflammasome markers in the liver. (B) Western blot images showing apoptotic markers in the liver. (C-G) Protein expression levels of inflammasome markers. (H-K) Protein expression levels of apoptotic markers. Results are expressed as mean ± SD (n = 3). (L) Representative plots showing Annexin V-FITC/PI staining. Bottom left quadrant, viable cells; bottom right quadrant, early apoptotic cells; upper right quadrant, late apoptotic cells. (M) Percentages of apoptotic cells, shown as the mean ± SD (n = 5). ^*^P-value < 0.05, ^**^P-value < 0.01, ^***^P-value < 0.001 compared with control; ^#^*P*-value < 0.05, ^##^*P*-value < 0.01, ^####^*P*-value < 0.0001 compared with TAA

As indicated by western blot analysis of different inflammasome components, we found that TAA injection-induced inflammasome activation in the liver (Figure 6A and C-F). An increased level of pyroptotic marker GSDMD also indicated the presence of pyroptosis (Figures 6A and 6G). However, not all of these protein expressions decreased following Sch B treatment. Only NLRP3 and GSDMD did the administration of Sch B decrease their expression. No change of caspase-1 expression was observed in Sch B-treated mice. Of note, IL-1β further increased by Sch B (Figure 6E and 6I). The expression of pro-apoptotic protein caspase-3 and caspase-8 also increased by TAA injection, whereas caspase-9 expression did not change. All of these caspase levels decreased at Sch B treatment (Figures 6B, 6H-6J). Anti-apoptotic protein BCL-2, on the other hand, showed a similar trend with caspase-3 (Figures 6B and 6K). We also analyze liver cell apoptosis by flow cytometry. As shown in Figures 6L and M, TAA injection increased liver cell apoptosis, which was reversed by Sch B.

*Analysis of inflammasome and apoptosis in the spleen*

**Figure 7:**
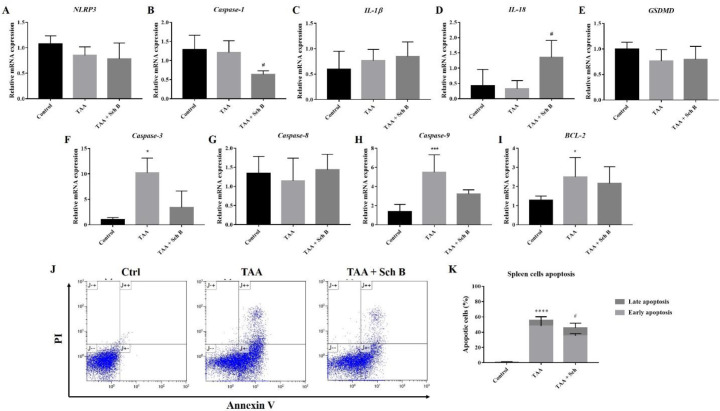
Analysis of inflammasome and apoptosis in the spleen. (A-E) Transcription levels of inflammasome markers in the spleen. (F-I) Transcription levels of apoptotic markers in the spleen. Results were presented as mean ± S.D. (n = 5). (J) Representative plots showing Annexin V-FITC/PI staining. (K) Percentages of apoptotic cells, shown as the mean ± SD (n = 5). ^*^P-value < 0.05, ^***^P-value < 0.001, ^****^P-value < 0.0001 compared with control; ^#^*P*-value < 0.05 compared with TAA

In the spleen, TAA injection resulted in only a slight decrease of *NLRP3*, *caspase-1*, *IL-18*, and *GSDMD*; and an increased *IL-1β *mRNA levels (Figures 7A-7E). However, no significant effect of Sch B on *NLRP3*, *IL-1β*, and *GSDMD* was observed, whereas it significantly decreased the level of *caspase-1* and increased the level of *IL-18* (Figures 7A-7E). We also found a significant increase in *caspase-3*, *caspase-9,* and *BCL-2* levels by TAA administration, which decreased by Sch B (Figures7F, 7H and 7I). *Caspase-8* levels were altered by neither TAA nor Sch B (Figure 7G). Sch B treatment also reduced the number of apoptotic cells caused by TAA (Figures 7J and 7K).

*Analysis of inflammasome and apoptosis in the kidney*

**Figure 8.**
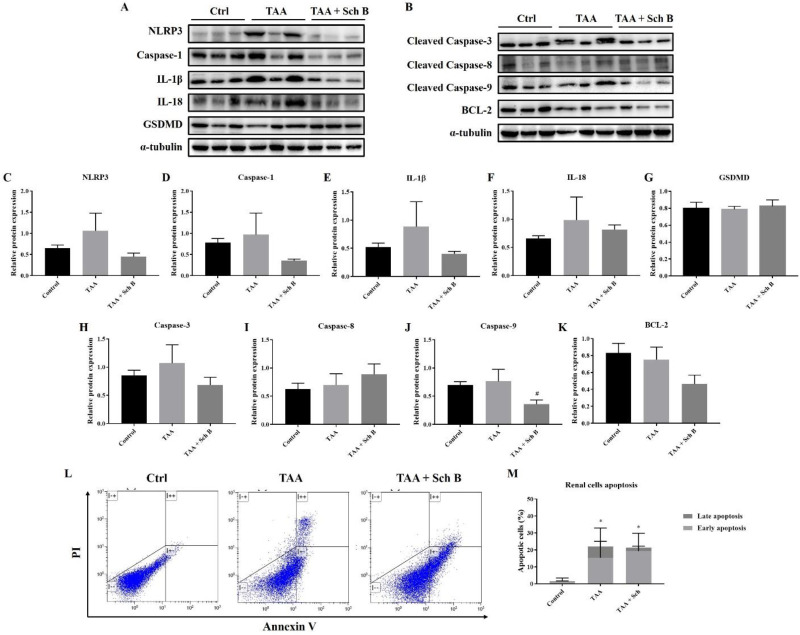
Analysis of inflammasome and apoptosis in the kidney. (A) Western blot images showing inflammasome markers in the kidney. (B) Western blot images showing apoptotic markers in the kidney. (C-G) Protein expression levels of inflammasome markers. (H-K) Protein expression levels of apoptotic markers. Results are presented as mean ± SD (n = 3). (L) Representative plots showing Annexin V-FITC/PI staining. (M) Percentages of apoptotic cells, shown as the mean ± SD (n = 5). ^*^P-value < 0.05 compared with control; ^#^*P*-value < 0.05 compared with TAA

TAA injection showed a significant NLRP3 inflammasome activation in the kidney, which was reduced by Sch B treatment (Figures 8A and 8C-8F). However, pyroptotic markers GSDMD did not change in levels (Figures 8A and 8G). TAA administration also increased the expression of apoptotic proteins. While the level of caspase-3 and caspase-9 reduced by Sch B, caspase-8 levels further increased by Sch B (Figures 8B and 8H-8J). Meanwhile, BCL-2 levels were decreased by TAA and even more significantly by Sch B (Figures 8B and 8K). These results were compatible to flow cytometric analysis. Despite the total apoptotic cells were not changed after Sch B treatment, the proportion of apoptotic cells shift from late apoptosis to early apoptosis (Figures 8L and 8M). 

*Analysis of inflammasome and apoptosis in the brain*

**Figure 9:**
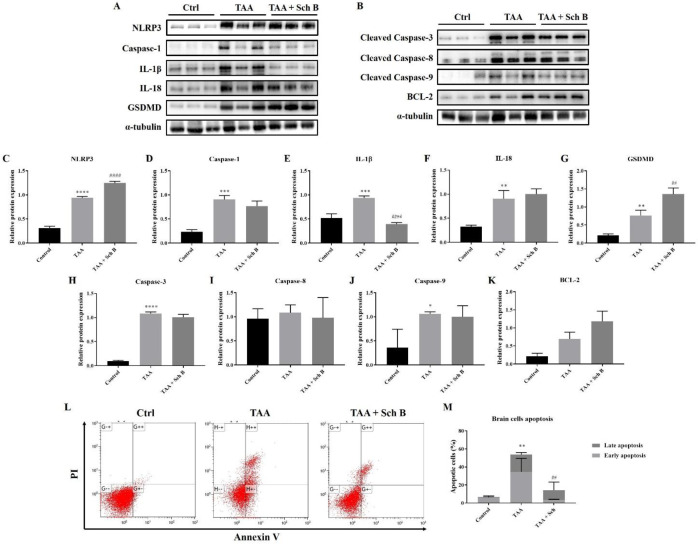
Analysis of inflammasome and apoptosis in the brain. (A) Western blot images showing inflammasome markers in the brain. (B) Western blot images showing apoptotic markers in the brain. (C-G) Protein expression levels of inflammasome markers. (H-K) Protein expression levels of apoptotic markers. Results are presented as mean ± SD (n = 3). (L) Representative plots showing Annexin V-FITC/PI staining. (M) Percentages of apoptotic cells, shown as the mean ± SD (n = 5). ^*^P-value < 0.05, ^**^P-value < 0.01, ^***^P-value < 0.001, ^****^P-value < 0.0001 compared with control; ^##^*P*-value < 0.01, ^####^*P*-value < 0.0001 compared with TAA

Western blot analysis of the brain showed increased inflammasome components by TAA administration. The use of Sch B resulted in a decreased caspase-1 and IL-1β expression but failed to lower the expression of NLRP3, IL-18, and GSDMD (Figures 9A and 9C-9G). Brain cell apoptosis significantly increased in TAA-injected mice, as shown by western blot analysis of apoptotic caspases (Figures 9B and 9H-9J) and flow cytometry (Figures 9L-9M). Although flow cytometric analysis revealed a decreased number of apoptotic cells, the expression of apoptotic caspases was not altered by Sch B treatment (Figures 9B and 9H-9J). On the contrary, anti-apoptotic protein BCL-2 increased by 1-fold and 2-fold after the mice were treated TAA or TAA and Sch B, respectively (Figures 9B and 9K). 

## Discussion

The induction of hepatitis or liver fibrosis by TAA has been shown to closely mimic the features of human liver diseases (26, 38). In this study, we used one single, high dose of TAA to induce acute hepatotoxicity in mice (26). We then administrated Sch B, a compound that was shown to be liver-protective (16-22), to these TAA-intoxicated mice. Our results showed that organ injuries occur severely in most tissues following TAA administration, and these injuries were differently associated with inflammasome activation and apoptosis. While Sch B treatment protected TAA-induced damages, it affected the inflammasome proteins and apoptotic proteins disparately.

In the liver, TAA resulted in the activation of NLRP3 inflammasome, which is essentially the same as that done by other groups (39, 40). Although Sch B treatment decreased the expression of NLRP3, it further increased the expression of IL-1β (Figure 6A, C-G). Since NLRP3 is the activator of caspase-1 (10), the decrease of NLRP3 and persist caspase-1 level suggest that Sch B selectively inhibits NLRP3 but not caspase-1 in the liver. The persisting caspase-1 facilitates the activation of inflammatory cytokines IL-1β and IL-18, but not the pyroptotic protein GSDMD. Accompanied with the decrease of LDH, which has also been used as a pyroptotic marker (31, 41), it suggests that liver pyroptosis is inhibited by Sch B. This inhibitory effect of Sch B has also been shown in other studies (42, 43), suggesting its potential mechanism in preventing inflammatory injuries. As we observed an improvement in liver histology following Sch B treatment (Figure 2), the significant increase of IL-1β may be explained by their properties of tissue regeneration and repair (8, 9 and 44). Meanwhile, TAA injection activates the extrinsic pathway of apoptosis in the liver, as suggested by an increased caspase-3 and caspase-8 level; and increased apoptotic cells. Sch B significantly reversed the apoptotic effects of TAA, with all caspase levels returning to normal (Figures 6H and 6I). These results indicate that Sch B improves TAA-induced liver injuries by downregulating the activation of the inflammasome and apoptotic caspases.

It appears that in the spleen, TAA did not activate the NLRP3 inflammasome. However, Sch B significantly downregulated *caspase-1* and upregulated *IL-18* levels (Figures 7A-7E). This leads us to hypothesize that another inflammasome activation may occur, altering these inflammasome effectors. However, there are currently no studies to investigate other inflammasome activation on splenic injuries. Further experimentation may be needed to prove our hypothesis. One study has suggested the role of Sch B in modulating splenic immune cells differentiation which prevents inflammation (45), this may also explain the improved pathology observed in this study (Figure 3). At the same time, Sch B was found to inhibit TAA-induced apoptosis (Figures 3B and 7F-7K). All these findings demonstrated that Sch B, through downregulating apoptotic caspases, can ameliorate splenic damages caused by TAA. 

In the kidney, our results indicated significant inflammasome activation and apoptosis by TAA injection (Figure 8). The involvement of inflammasome or apoptosis is also shown in other acute or chronic kidney diseases (40, 46). Upon Sch B treatment, inflammasome activation was inhibited and the proportion of apoptotic cells shifted from late apoptosis to early apoptosis, which leads to a reversion of kidney damages (Figure 3; Table 2). These results bear a close resemblance to nephrotoxicity caused by mercury (23) or cyclosporine A (32), which all suggested a beneficial effect of Sch B. These observations put forward to that Sch B is able to reverse TAA-induced kidney damages through downregulating inflammation and apoptosis.

Lastly, TAA significantly increased inflammasome components and apoptotic caspases in the brain (Figures 9A-9J), suggesting a pathophysiological change. Although we did not observe any specific damages in the brain of TAA-treated mice (except in one brain section there was a marked area of perivascular infiltration; Figures 4E and 4F), we found an altered BBB permeability, as indicated by the increase of albumin and total protein ratio (47). Following Sch B treatment, these ratios decreased (Table 2). Although the increase of CSF total protein may not always mean BBB damage as CNS can also synthesize immunoglobulins and other proteins (48, 49), albumin synthesis does not occur in the CNS; therefore, albumin present in CSF usually derives from plasma (50, 51). We, therefore, suggest the recovery of BBB permeability by Sch B. Notably, Sch B did not result in the decrease of inflammasome component or pro-apoptotic caspases. Although the increase of BCL-2 may be involved in reducing brain cell apoptosis, Sch B may also have targeted another pathway to ameliorate TAA-induced brain damages, which will require further investigation. 

**Table 1:** Primer pairs that were used in this study

Gene name	Primer pairs (5'-3')	GenBank Accession
β-actin	Forward-GTGACGTTGACATCCGTAAAGA	NM_007393
	Reverse-GCCGGACTCATCGTACTCC	
NLRP3	Forward-ATTACCCGCCCGAGAAAGG	NM_145827
	Reverse-CATGAGTGTGGCTAGATCCAAG	
Caspase-1	Forward-ACAAGGCACGGGACCTATG	NM_009807
	Reverse-TCCCAGTCAGTCCTGGAAATG	
IL-18	Forward-GTGAACCCCAGACCAGACTG	NM_008360
	Reverse-CCTGGAACACGTTTCTGAAAGA	
IL-1β	Forward-GAAATGCCACCTTTTGACAGTG	NM_008361
	Reverse-TGGATGCTCTCATCAGGACAG	
GSDMD	Forward-GTGTGTCAACCTGTCTATCAAGG	NM_026960
	Reverse-CATGGCATCGTAGAAGTGGAAG	
Caspase-3	Forward-CTCGCTCTGGTACGGATGTG	NM_009810
	Reverse-TCCCATAAATGACCCCTTCATCA	
Caspase-8	Forward-TGCTTGGACTACATCCCACAC	NM_009812
	Reverse-GTTGCAGTCTAGGAAGTTGACC	
Caspase-9	Forward-GGCTGTTAAACCCCTAGACCA	NM_015733
	Reverse-TGACGGGTCCAGCTTCACTA	
BCL-2	Forward-GCTACCGTCGTGACTTCGC	NM_177410
	Reverse-CCCCACCGAACTCAAAGAAGG	

**Table 2:** Biochemical analysis

**Group**	**BIOCHEMISTRY**										
	**AST(U/L)**	**ALT(U/L)**	**LDH(U/L)**	**CREA(mg/dL)**	**BUN(mg/dL)**	**S.TP(g/dL)**	**S.ALB(g/dL)**	**C.TP(g/dL)**	**C.ALB(g/dL)**	**CSF/SERA.TP**	**CSF/SERA.ALB**
Control	88.24 ± 6.65	64.29 ± 21.43	225.54 ± 45.99	0.55 ± 0.18	20.50 ± 2.78	9.02 ± 0.40	3.20 ± 0.39	0.32 ± 0.04	0.26 ± 0.04	0.04 ± 0.01	0.08 ± 0.02
TAA	621.54 ± 217.02^*^	498.25 ± 133.69^*^	1653.12 ± 678.69^*^	1.74 ± 0.28^*^	66.94 ± 14.80^*^	8.50 ± 0.85	2.53 ± 0.30	1.30 ± 0.61	0.66 ± 0.08^*^	0.16 ± 0.07^*^	0.26 ± 0.05^*^
TAA+Sch B	416.20 ± 84.68	346.00 ± 233.46	834.17 ± 309.39^#^	1.18 ± 0.33 ^#^	45.54 ± 12.15^#^	8.33 ± 0.58	3.04 ± 0.33	1.15 ± 0.41	0.49 ± 0.11	0.14 ± 0.04	0.16 ± 0.02^#^

AST: aspartate aminotransferase; ALT: alanine aminotransferase; LDH: lactate dehydrogenase; CREA: creatinine; BUN: blood urea nitrogen; S.TP: serum total protein; S.ALB: serum albumin; C.TP: CSF total protein; C.ALB: CSF albumin Data are expressed as mean ± SD (n = 3 control mice; n = 5 TAA-injected mice; n = 5 Sch B-treated mice).

^*^ *P*-value < 0.05, statically significant when compared with control group.

^#^ *P*-value < 0.05, statically significant when compared with the TAA group.

## Conclusion

Collectively, our findings reveal a beneficial effect of Sch B on different organ damages in a TAA-induced acute hepatitis mice model. Sch B provides this protective effect through modulation of inflammasome activation and apoptosis. Further investigation of the immune-regulatory effect of Sch B may provide additional insights into the treatment of liver disease. 

## Conflict of Interest

The authors declare no conflict of interest.

## Author contributions

H.Y.P.L and S.Y.P. conceived and designed the experiments; H.Y.P.L., M.Y.H., and T.R.L. performed the experiments and analyzed the results; H.Y.P.L. wrote the paper. 
